# Freeze-Thawing Treatment as a Simple Way to Tune the Gel Property and Digestibility of Minced Meat from Red Swamp Crayfish (*Procambarus clarkiix*)

**DOI:** 10.3390/foods11060837

**Published:** 2022-03-15

**Authors:** Tao Ye, Xing Chen, Yajun Zhu, Zhina Chen, Yun Wang, Lin Lin, Zhi Zheng, Jianfeng Lu

**Affiliations:** 1 School of Food and Biological Engineering, Hefei University of Technology, Hefei 230009, China; yetao430@163.com (T.Y.); zyjdyx2@mail.hfut.edu.cn (Y.Z.); linlin@hfut.edu.cn (L.L.); zhengzhi@hfut.edu.cn (Z.Z.); 2 School of Bioengineering, Huainan Normal University, Huainan 232038, China; chenzhina0625@163.com (Z.C.); wy2015@ustc.edu.cn (Y.W.); 3 State Key Laboratory of Food Science and Technology, Jiangnan University, Wuxi 214122, China; xingchen@jiangnan.edu.cn; 4 Key Laboratory for Agricultural Products Processing of Anhui Province, Hefei University of Technology, Hefei 230009, China; 5 Engineering Research Center of Bio-Process, Ministry of Education, Hefei University of Technology, Hefei 230009, China

**Keywords:** freezing, thawing, crayfish, meat yield, gel properties, digestibility, antioxidant activity

## Abstract

The effects of freezing methods, including rapid freezing (RF) or slow freezing (SF), combined with thawing methods, e.g., water immersing thawing (WT) or cold thawing (CT), on the meat yield, drip loss, gel properties, and digestive properties of meat detached from red swamp crayfish were investigated. RF greatly reduced the freezing time compared to SF, and the thawing time of frozen crayfish was obviously shortened by WT in comparison to CT. RF and CT improved the meat yield but increased the drip loss, probably as a result of the greater protein denaturation or degradation. A soft and flexible gel was obtained by SF-CT, while a hard one was achieved by RF-WT. An SEM analysis showed that SF resulted in rough and irregular microstructures with larger pore sizes. Freeze-thawing led to an increase in the β-sheet content at the expense of α-helix and variations in the microenvironment of tyrosine and tryptophan residues in protein molecules of the gels, which was more pronounced in the SF-CT group. Moreover, freeze-thawing could cause enhanced protein digestibility but reduce the antioxidant activity of gels. These findings underline the promise of the freezing-thawing treatment in tuning the gel-based meat products of crayfish.

## 1. Introduction

Aquatic products are among the most important foods consumed worldwide due to their presence of high-value proteins, essential micronutrients, and desirable sensory properties (delicious taste and unique aroma) [1]. However, these valuable food ingredients are highly perishable after harvest, hence their preservation becomes particularly crucial for the aquatic processing industry [2]. Freezing, as a common unit operation of food processing, can effectively reduce the water activity, inhibit microbial growth and enzyme activity, and retard the quality deterioration, thereby extending the shelf-life of aquatic products [3,4].

The ice crystals formed by freezing are disruptive to muscle fibers and cells of seafood, and they can induce protein denaturation, resulting in thawing drip loss and the deterioration of protein functional properties [5,6]. Interestingly, the freezing-induced protein denaturation could be applied to weaken (or loosen) the connection tissue between the shell and the meat, thus facilitating the subsequent shucking of shrimp [7]. Data from previous research suggested that a freeze-thawing treatment prior to peeling was beneficial to improving meat yield and organoleptic quality, as well as reducing the energy or labor consumption required for the complete removal of the shell from the meat [8].

The red swamp crayfish (*Procambarus clarkii*) is an important economic species in China with high market value because of its nutritional attributes (high protein and low-fat content) and ease of breeding (fast growth rate and excellent adaptability) [9]. In 2020, the annual cultivation amount of red swamp crayfish was close to 2.4 million tons, which was an increase of about 14.55% compared to 2019 [10]. The output of the processed crayfish was also continually increasing as a response to the climbing demand. Though several deep-processing products, such as the frozen boiled crayfish tail, frozen crayfish meat, and the ready-to-cook seasoned crayfish, have been developed, they still cannot satisfy the needs of consumers for diversified products [11]. Nowadays, developing meat gel products from crayfish meat is increasingly attracting the attention of aquatic processing enterprises and consumers [12].

Meat separation is the essential stage for gel preparation. Its pretreatment method plays an important role in the subsequent deep processing of crayfish. Our preliminary experiments proved that freeze-thawing was effective for the shelling of crayfish. While freezing weakens the connected tissue, it will inevitably have some negative effects on the separated muscle, such as drip loss, freezing-induced protein denaturation, and decreased gelling properties [6]. Moreover, the typical thawing methods (thawed by cold water or air) are also crucial in the quality control of meat products after frozen treatment [13]. However, to our best knowledge, few studies have investigated the impact of freeze-thawing on the meat yield and gelling properties of the shucked meat from crayfish.

Therefore, in this study, the effects of freezing methods, namely, liquid nitrogen rapid freezing (RF) and slow freezing (SF), combined with two thawing methods, including water-immersing thawing (WT) and cold thawing (CT), on meat yield and heat-induced gel properties of red swamp crayfish were investigated. The gel properties, including gel strength, deformation, and breaking force, color parameters, water-holding capacity, microstructure, protein secondary structural change, as well as the protein in vitro digestibility and antioxidant activity, were comprehensively evaluated to elaborate on the relationship between the gel properties and freezing-induced protein denaturation.

## 2. Materials and Methods

### 2.1. Materials

Live crayfish (*P. clarkii*) with weight of 15.93 ± 0.52 g were purchased in March 2021 from a local aquatic products market (Anhui, China) and were immediately transported to the food processing laboratory within 1 h. The crayfish (*n* = 75) were kept alive at about 10 °C, and the subsequent experiments for crayfish were proceeded within 24 h.

### 2.2. Freezing and Thawing Treatments

The live crayfish (*n* = 75) were randomly distributed into five clusters and were either (1) frozen by liquid nitrogen rapid freezing (RF), and thawed by water immersing thawing (WT) at about 10 °C, RF-WT; or (2) frozen by refrigerator slow freezing (SF) by using a BCD-216SDN refrigerator (Haier Co., Ltd., Shandong, China) at −18 °C, and thawed by WT, SF-WT; or (3) frozen by RF, and thawed by cold thawing (CT) in a BCD-216SDN refrigerator at 4 °C, RF-CT; or (4) frozen by SF and thawed by CT, SF-CT. The unprocessed crayfish were served as the control group, CG. The center temperature of crayfish treated by different freezing-thawing treatments was recorded by a multi-channel temperature logger (Model AT4202, Anbai Instrument Co., Ltd., Changzhou, China) with a precision of ±0.2 °C.

### 2.3. Crayfish Meat Collection, Meat Yield, and Drip Loss Measurement

After the above freezing-thawing treatment, the crayfish muscle tissue was detached from the shells manually. The meat yield of crayfish (*Y*) was defined as the mass ratio of obtained crayfish meat (*W*_1_) to crayfish (*W*_0_) and calculated as Equation (1).

*Y* = *W*_1_/*W*_0_ × 100% (1)

Drip loss was defined as a percentage of weight loss of crayfish meat after shelled and expressed as Equation (2).

*Drip loss* = (*W*_0_ − *W*_1_ − *W*_2_)/*W*_0_ × 100% (2)

where *W*_0_ is the weight of five fresh crayfish of each group, *W*_1_ is the weight of the detached crayfish meat, and *W*_2_ is the weight of the shell and head after manual peeling.

### 2.4. Gel Preparation

The peeled crayfish meat was chopped at 5000 rpm for 3 min by using a MQ5035 blender (Braun Co., Ltd., Kronberg, Germany). Afterward, 2% (*w*/*w*) of NaCl was added to the paste and blended for 1 min at 1000 rpm for protein dissolution. The mixture was stuffed into beaker of 5 mL. Then the packed beakers were heated in a water bath (95 °C, 6 min), followed by cooling for 30 min. The crayfish meat gels were stored at 4 °C before further use.

### 2.5. Mechanical Properties and Color Analysis of Gels

The mechanical properties, including gel strength (g × mm), breaking force (g), and deformation (mm) of crayfish meat gel samples (about 15 mm in diameter and 15 mm in height.) were analyzed by using a TA. XT plus texture analyzer (Stable Micro System Co., London, UK). The conditions were selected as follows: probe type, P/0.25S; test speed, 1.0 mm/s; pre-test speed, 1.0 mm/s; post-test speed, 5.0 mm/s; trigger force, 5 g; pressing distance, 12 mm. At least 6 samples were analyzed for each group. The color of crayfish meat gels was estimated by a colorimeter (Model NR60CP, Sanenshi Co., Ltd., Guangdong, China), and the test conditions were as follows: observer: CIE 10° standard observer; illuminant: D_65_; measuring time: 0.4 s.

### 2.6. Water-Holding Capacity Determination and SEM Observation

Water-holding capacity (WHC) of gels of crayfish meat was determined following the method of Liang, et al. [14]. The crayfish meat gels were cut into cylindrical flakes with 2 mm thickness and were submerged in glutaraldehyde solution (2.5%, *v*/*v*) at 4 °C for 24 h, then dehydrated with a series of ethanol solutions (60–100%). After substituting the ethanol with isoamyl acetate, the frozen gels were freeze-dried for 4.5 h in a model FD-ID-50 freeze dryer (Bilang Instrument Co., Ltd., Shanghai, China). The freeze-dried samples were sputter-coated, and the microstructure of crayfish meat gels was detected using a Carl Zeiss Microscope (Oberkochen, Germany) at voltage of 3.0 kV.

### 2.7. Ultraviolet (UV) Spectrum and Fourier Transform Infrared Spectroscopy (FTIR) Analysis

The crayfish meat gels were diluted to 0.5 mg/L with 0.6 mol/L KCl solution, and the UV-Vis absorption spectrum at wavelength of 220–320 nm was recorded by D-7PC spectrophotometer (Feile Instrument Co., Ltd., Nanjing, China). The second-derivative UV spectra was calculated and plotted by Origin 2018 software. For the FTIR analysis, the crayfish meat gels were frozen and then freeze-dried for 6 h by a freeze dryer (Model FD-ID-50, Bilang Instrument Co., Ltd., Shanghai, China). The freeze-dried meat gels were blended with potassium bromide of spectral purity, and the mixture was compressed into cylindrical sheet. The spectra of crayfish meat gels were measured in a Nicolet 6700 spectrometer (Thermo Fisher Scientific Co., Waltham, MA, USA). PeakFit software was applied to gain the protein secondary structures [15].

### 2.8. In Vitro Gastrointestinal Digestion and Antioxidant Activity of Crayfish Meat Gels

In vitro gastrointestinal digestion of the crayfish meat gels was performed by the method of Singh, et al. [16] with some modifications. Crayfish meat gels (1 g) were homogenized with phosphate buffer (7 mL, pH 2.0). After the homogenate was pre-incubated at 37 °C for 5 min, pepsin solution (1.5 mg/mL, 0.5 mL) was added and then the hydrolytic reaction was performed in a water bath at 37 °C for 60 min. After the simulated gastric digestion, the mixture was added with 7.5 mL of 0.5 mol/L Tris HCl to adjust the pH to 6.9. Then, 0.5 mL of trypsin solution (1.5 mg/mL) was mixed, and the digestion reaction was conducted for 60 min at 37 °C. The reaction was terminated in boiling water bath for 5 min, and the reactant was centrifuged at 5000 rpm for 20 min at 4 °C to collect the supernatant for testing. Antioxidant activities of the simulated digested crayfish meat gels were measured according to the previous literature. The DPPH radical scavenging activity (DPPH-RSA) was assessed by the method of [17]. Ferric reducing antioxidant power (FRAP) and ABTS-radical scavenging activity (ABTS-RSA) were determined according to the procedures of Singh, Prabowo, Benjakul, Pranoto, and Chantakun [16].

### 2.9. Protein Patterns of Gels before and after Digestion

SDS-PAGE was analyzed using the method of Li, et al. [18] with some modifications. Before digestion, crayfish meat gels (4 g) were mixed sodium dodecyl sulfate (SDS) solution (1%, *w*/*v*, 36 mL) and homogenized (5000 rpm, 3 min) with a T18 homogenizer (IKA, Schwarzwald, Germany). The mixture was centrifuged (5000 rpm, 5 min, 6 °C) by a 5430R centrifuge (Eppendorf, Hamburg, Germany), and the supernatant was separated and adjusted to a uniform protein concentration level. After digestion, 0.4 mL of the supernatant from the digested sample was used for analysis. Electrophoresis was performed by a DYCZ-24KF electrophoresis apparatus (Liuyi Biological Technology Co., Ltd., Beijing, China) at 170 V for about 3 h.

### 2.10. Statistical Analysis

All data were statistically analyzed by using the SPSS 20.0 software (IBM, Armonk, NY, USA) by Duncan’s multiple range test at a significance level of *p* < 0.05. A minimum of 3 batches of samples were used for the test.

## 3. Results and Discussion

### 3.1. Freezing and Thawing Curves of Crayfish

**Figure 1:**
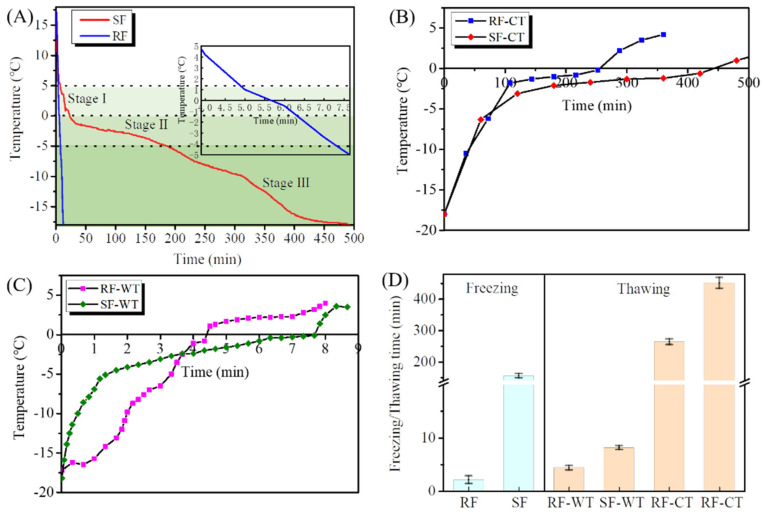
Freezing curves (**A**), refrigerator thawing curves (**B**), water immersing thawing curves (**C**), and freezing-thawing time (**D**) of red swamp crayfish. RF, rapid freezing; SF, slow freezing; CT, cold thawing; WT, water immersing thawing.

The freezing curves of crayfish subjected to SF and RF are compared in Figure 1A. The time required for SF and RF was 490 min and 13 min, respectively, as the core temperature of the crayfish decreased from 17.2 °C to −18 °C. As expected, RF shortened the total freezing time by about 97.34%, compared with SF. The freezing rate could be defined as rapid (fast) freezing when the time for the core temperature of food to traverse from −1 to −5 °C is less than 30 min, and it is considered slow freezing once the time exceeds 30 min [19]. The freezing methods, SF and RF, allowed the core temperature to decrease from −1 °C to −5 °C in 156.33 and 2.17 min, respectively (Figure 1D), indicating that SF and RF in the current study belonged to typical slow freezing and rapid freezing, respectively. Freezing rate is the main factor affecting the ice crystal size in the food freezing process [20]. A small number of large extracellular ice crystals could be formed by slow freezing, whereas small ice crystals distributed both intra- and extra-cellularly are produced by fast freezing [21].

Generally, the freezing curve of crayfish can be divided into three stages, i.e., pre-freezing stage (Stage I, 5~0 °C), phase transition (Stage II, 0~−5 °C), and sub-freezing (Stage III, −5~−18 °C). Among those stages, stage II was considered the maximum-ice-crystal formation zone, which is critical for the size and distribution of ice crystals in frozen crayfish [22]. As shown in Figure 1A, the RF greatly reduced the elapsed time of this stage as compared with the SF. It was suggested that RF shorten the time to pass through the maximum-ice-crystal formation zone by accelerating the freezing rate. Surface energy theory could explain this advantage of RF, i.e., smaller ice crystals have higher surface energy due to the smaller radius of curvature and larger surface-to-volume ratio, which is thermodynamically unstable, and freezing tends to reduce the surface energy by increasing the size of the crystal, thus providing the driving force for the ice crystal growth. This could be conducive to promoting a more rapid phase transition and reducing the freezing time [23].

The frozen crayfish were thawed by CT and WT until the core temperature reached 4 °C. The CT and WT curves of crayfish frozen by RF and SF are shown in Figure 1B,C respectively. The thawing times of CT for the RF and SF groups were 264 min and 451 min, respectively, while the thawing times of WT for those groups were 4.41 min and 8.17 min, respectively (Figure 1D), indicating that WT greatly shortened the thawing time of the frozen crayfish. Similar results were also found in the thawing process for the frozen bighead carp (*Aristichthys nobilis*) fillets [24], as well as the frozen pompano (*Trachinotus ovatus*) [25]. This might be explained by the fact that the water [0.604 W/(m•K)] has a higher thermal conductivity (i.e., heat transfer rate) than air [0.066 W/(m•K)] [19,21]. Likewise, it was also found that SF increased the thawing time of crayfish compared to RF, irrespective of thawing by CT or WT, probably owing to the relatively high freezing point of ice crystals in the intercellular space [19].

### 3.2. Meat Yield and Drip Loss of Crayfish Meat

**Figure 2:**
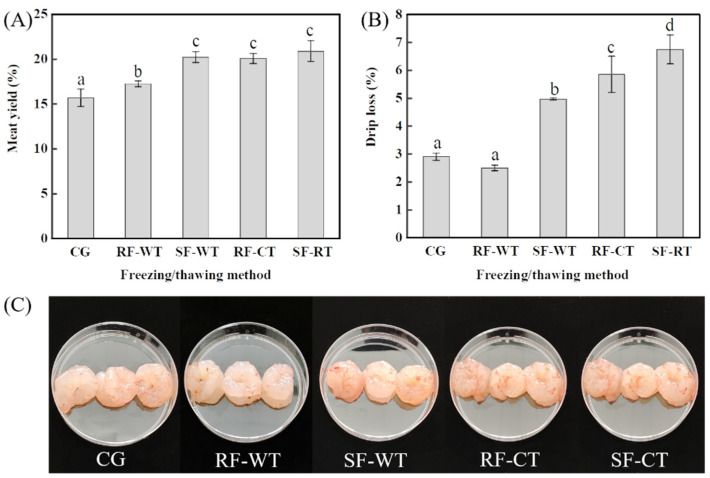
Meat yield (**A**), drip loss (**B**), and appearance (**C**) of crayfish meat treated by different freezing-thawing combinations. Different letters (a–c) in (**A**) for meat yield and letters (a–d) in (**B**) for drip loss indicate significant differences (*p* < 0.05). CG, control group; RF, rapid freezing; SF, slow freezing; CT, cold thawing; WT, water immersing thawing. *n* = 15.

As shown in Figure 2A, the meat yield of crayfish in the CG group was 15.68%. After RF-WT, SF-WT, RF-CT, and SF-CT treatments, the yield significantly increased to 17.24%, 20.22%, 20.06%, and 20.88%, respectively (*p* < 0.05), indicating that the freeze-thaw process was capable of improving the meat yield. The muscle of crayfish is closely connected to the shell through connective tissue (epidermis, in which protein is the main component) [26]. The freeze-thawing treatment allowed the protein of connective tissue to denature, resulting in shell loosening, which facilitated the subsequent peeling [7]. Within a certain range, the greater the degree of protein denaturation, the easier it is to separate the meat from the shell, and the better the integrity of the detached meat (Figure 2C). Thus, the yield of crayfish meat can be accordingly increased. In addition, the meat yield in the SF-WT, RF-CT, and SF-CT groups was significantly higher than that of RF-WT (*p* < 0.05), implying that the protein denaturation in the RF-WT group was the lowest compared to the other treatments.

Changes in drip loss of crayfish samples treated by freeze-thawing are shown in Figure 2B. The drip loss of CG was 2.89 ± 0.13%, and freeze-thawing treatments of SF-WT, RF-CT, and SF-CT significantly increased the drip loss to 4.96–6.74% (*p* < 0.05). Evidently, crayfish frozen by SF exhibited higher drip loss than that of RF samples (*p* < 0.05). Thawing drip loss is related to the disruption of muscle fibers and cells by the mechanical stress of ice crystals (volume expansion) as well as the protein denaturation caused by pH changes during freezing [5]. The freezing rate affects the size and distribution of ice crystals in the muscle of the frozen crayfish. As the freezing rate increased, the size of ice crystals became smaller and their distribution narrower, leading to less damage to the muscle fibres and proteins, which could reduce the drip loss of the meat after thawing [27,28]. Therefore, SF and CT seemed to denature the protein to a greater extent during the freeze-thawing process, thus increasing the meat yield, as well as the drip loss.

### 3.3. Texture and Color of Crayfish Meat Gels

**Figure 3:**
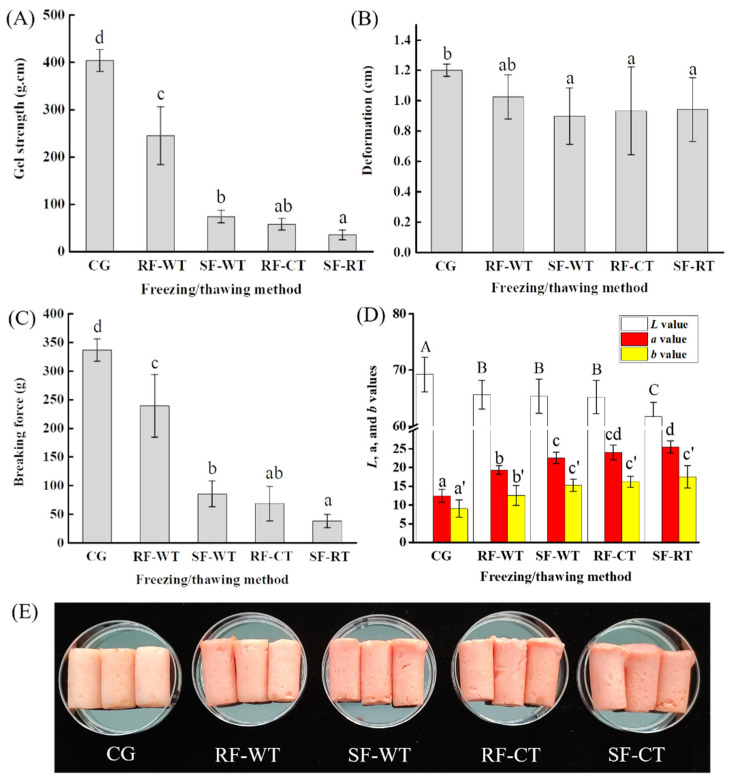
Texture properties (**A**–**C**), color (**D**), and appearance (**E**) of crayfish meat gels prepared by different freezing-thawing combinations. Different letters (a–d) in (**A**) for gel strength, letters (a–b) in (**B**) for deformation, letters (a–d) in (**C**) for breaking force, and letters ((**A**–**C**) for L value; (a–c) for a value; a’–b’ for b value) in (**D**) indicate significant differences (*p* < 0.05). CG, control group; RF, rapid freezing; SF, slow freezing; CT, cold thawing; WT, water immersing thawing.

The gel strength, deformation, and breaking force of crayfish meat gels as influenced by freeze-thawing treatments are shown in Figure 3. The gel strength of the RF-WT, SF-WT, RF-CT, and SF-CT groups was 244.56, 73.26, 57.30, and 34.67 g•mm, respectively, which were all inferior to the control group (403.58 g•mm) (*p* < 0.05). As shown in Figure 3C, the decrease in gel strength can be reflected by the obvious reduction in breaking force, whereas the variations in the deformation were relatively small (Figure 3B). This revealed that the crayfish subjected to the freeze-thawing pretreatment resulted in a soft and flexible texture for subsequent heat-induced gels, which might be beneficial to the people suffering from dysphagia [17].

Moreover, it was found that crayfish meat gels with varied gel strengths could be obtained by different freezing and thawing combinations. In terms of thawing by WT, the gel strength of RF samples (244.56 g•mm) was significantly greater (*p* < 0.05) than that of the SF (73.26 g•mm). For the CT, the gel strength of RF samples (57.30 g•mm) was also higher than that of SF (34.67 g•mm), though the difference is statistically insignificant (*p* > 0.05) (Figure 3A). Therefore, RF was found to be in favor of maintaining the gel strength of crayfish meat compared with SF. Additionally, the gel strength of WT samples was significantly higher (*p* < 0.05) than that of the CT by the same freezing method (RF or SF). In conclusion, RF and WT could preserve the mechanical properties of the crayfish meat gels compared to SF and CT.

Li, Wang, Kong, Shi, and Xia [6] reported that the gel strength of myofibrillar protein (MP) gels from mirror carp (*Cyprinus carpio*) significantly decreased from 2.44 N to 1.94 N when subjected to a freeze-thawing process (frozen in a refrigerator at −25 °C and thawed in a refrigerator at 4 °C for 12 h). Subsequent trials found that the freeze-thawing caused MP denaturation and aggregation, as evidenced by the reduced protein solubility and Ca^2+^-ATPase activity, the increased particle size, and the high molecular weight of MP, as well as the increased average roughness of the MP film (Rg, atomic force microscopy), leading to the reduced gel-forming ability. A previous study on the MP gelling properties of mirror carp treated with freeze-thawing (frozen at −25 °C and thawed at 4 °C for 12 h) found that freeze-thawing decreased the storage modulus G’ of MP gels, and the ζ-potential of MP declined from 26.87 mV to 23.9 mV, while the median diameter (*d*_V,0.5_) of protein increased from 24.41 μm to 32.3 μm, implying protein aggregation of MP occurred during freeze-thawing [29]. Consequently, changes in the functional and structural properties of MP are responsible for the decreased gelling properties.

The freezing-induced denaturation of proteins could be ascribed to the ice crystal formation, freeze-concentration, and cold denaturation of proteins. Among those factors, protein cold denaturation was considered as the spontaneous unfolding of protein as a result of the exothermic process of decreased entropy during cooling, which could be almost negligible compared with the other two factors [5]. Qian, Hu, Mehmood, Li, Zhang, and Blecker [28] compared the effects of slow freezing (SF, frozen at −20 °C for 12 h) and ultra-fast freezing (UFF, frozen at −80 °C for 12 h) on the size and distribution of ice crystals in bovine Longissimus dorsi muscles, and they found that the average number, the equivalent diameter, and the ratio of ice crystals sum area to that of cell area of SF samples were about 116, 35 μm, and 0.32, respectively, while those indexes of UFF samples were about 169, 25 μm, and 0.15, respectively, suggesting that a larger number of ice crystals of smaller size were formed by UFF. The freezing rate (slow and fast freezing) is largely related to protein denaturation, and MP denaturation was more pronounced in slow compared to fast freezing during the freezing of pork steaks [27].

Although the mechanism of the impact of ice crystal formation on MP denaturation is currently not well disclosed, several clues are available for better understanding. During the process of freezing, the extracellular water begins to freeze, and the unfrozen water within the cells will migrate to the outside of the muscle fibers to form extracellular ice crystals. Then muscle fibers are severely compressed by the extracellular ice crystals, leading to the distortion of rigor bonds (actomyosin cross-bridges) and thereby the denaturation of myosin heads [1]. This could also bring about the increased concentrated solutes of the unfrozen water phase and decrease the pH around the structural proteins (actin and myosin filaments), resulting in freezing-induced denaturation of MP stemming from the higher ionic strength and lower pH [20]. Fast freezing might reduce the exposure time of myofilaments to concentrated solutes, alleviating the pH variation, probably through trapping part of the protons within the small ice crystals, as well as the fiber compression, and thus reducing the protein denaturation [1,27]. Besides, the protein oxidation caused by the ice-crystal growth might also disrupt the protein structure, and irregular ice crystals produced by slow freezing could promote the MP oxidation [6].

In fact, the thawing process is another crucial component affecting the gelling properties of frozen meat [13], because it can cause physical damage to the muscle cells and denaturation, oxidation, and aggregation of MP [1,30]. For the aquatic product, during the thawing process, the effects of the action of endogenous enzymes (such as cathepsin L) released from the distorted cells [31] and the metabolism of rejuvenated microorganisms [30] on the MP are non-ignorable. In this study, the gel strength of WT samples was superior to that of CT, and this might be ascribed to the greatly reduced thawing time of WT (Figure 1D), which decreased the action time of microorganisms and enzymes.

The color parameters (*L*, *a*, and *b*) of crayfish meat gels prepared from freeze-thawed crayfish were displayed in Figure 3D. A freeze-thawing cycle of crayfish prior to gel preparation led to a significant decrease in the *L* values of the subsequent heat-induced gels (*p* < 0.05), compared with CG (untreated crayfish). Previous literature reported that the high value of lightness in gels could be related to protein aggregation, and the increased cross-links promote a compact structure, leading to a greater area of light reflectance [32]. The decrease in the whiteness of protein gels from mirror carp was due to the freezing-induced denaturation of MPs, and protein denaturation could reduce the free water content in the gel network structure, which decreased the reflection [6]. However, freeze-thawing treatments significantly improved the *a* and *b* values of crayfish meat gels (*p* < 0.05), making the gels appear attractive red, and this is consistent with the visual appearance as presented in Figure 3E. This could benefit from the more pigment cells (containing astaxanthin) separated from the epidermis as a result of the freezing-induced denaturation of connective tissue [7,26], and the result was consistent with the improved meat yield of crayfish observed in Figure 2A. Hence, the crayfish meat gels with various gel strengths can be prepared by different freezing-thawing combinations, and the color of the gels could be ameliorated by the freeze-thawing process.

### 3.4. WHC and SEM of Gels

**Figure 4:**
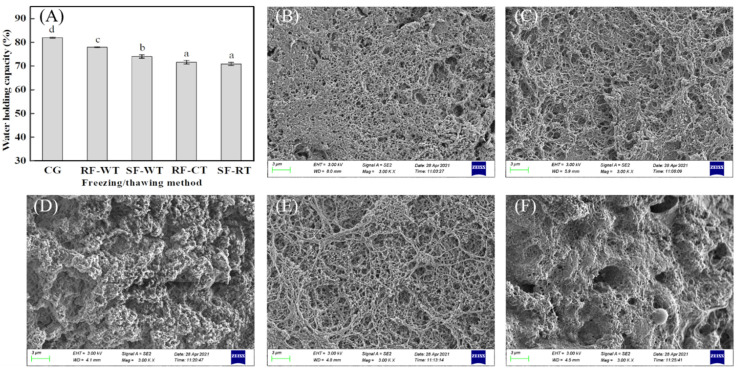
Water-holding capacity (**A**) and scanning electron microscopic images (**B**–**F**) of heat-induced gels of crayfish meat processed by several freeze-thawing combinations. (**B**): CG; (**C**): RF-WT; (**D**): SF-WT; (**E**): RF-CT; (**F**): SF-CT. CG, control group; RF, rapid freezing; SF, slow freezing; CT, cold thawing; WT, water immersing thawing. Different letters (a–d) in (A) for water-holding capacity indicate significant differences (*p* < 0.05).

The impact of the freeze-thawing process on the WHC of gels was shown in Figure 4A. The WHC of the gels made from the freeze-thawed crayfish meat varied from 70.85–77.92%, while the value of the control was 81.96%, indicating that the freeze-thawing treatments caused a significant decrease in the WHC of gels (*p* < 0.05). Cando, et al. [33] reported that the WHC of surimi gels was associated with the type and number of water-protein interactions, in addition to the intrinsic network structure within the gels. A previous study on the gel properties of MP from mirror carp (*C. carpio*) treated with freeze-thaw cycles found that the immobilized water (*T*_21_ water), trapped in the microstructure of gels, was the major component in the gels, and its relaxation time became longer after freeze-thawing, suggesting that the amount of free and mobilized water in MP gels increased after a freeze-thawing treatment. This might be due to the loose and porous structure of MP gel because of the freezing-induced denatured proteins [6].

As shown in Figure 4B–F, the gels in the CG and RF-WT groups exhibited a compact gel network with small regular pores (Figure 4B,C), revealing that RF-WT was beneficial to the microstructural maintenance of crayfish meat gels, and this was in accordance with the relatively good mechanical properties observed in Figure 3A–C. However, RF combined with CT (RF-CT group) resulted in a slightly porous structure (Figure 4E). Since the longer thawing time (264 min vs. 4.41 min) could endow the endogenous enzymes and microorganisms with the ability to rejuvenate and act on the proteins, leading to the degradation of MPs [30]. Yang, et al. [34] reported that the TVB-N values of frozen swimming crab (*Portunus trituberculatus*) significantly increased from 6.67 mg/100 g (fresh meat) to 8.12 mg/100 g and 7.53 mg/100 g, respectively, after air thawing (at 15 °C for 270 min) and water-immersing thawing (at 15 °C for 101 min). Interestingly, slow freezing rendered the gel network rough and irregular (Figure 4D,F), especially for the SF-CT group, the reticular structure was seriously damaged (Figure 4F). The microstructure of crayfish meat gels was shown to be more negatively affected by the SF, which could be the result of the freezing-induced denaturation of MPs (as discussed in Section 3.3).

Generally, MPs’ gelation contains the following two major stages: (i) MPs dissolve to form protein sol under the action of salt (2~3% sodium chloride can usually realize the sufficient dissolution of MPs), comprising of myosin and actin separation and depolymerization of myosin chains; (ii) the dissociated MPs undergo thermal conformational changes and aggregations under conditions of heat. The stage ii is probably as follows: Myosin S1 expands when the temperature rises to around 35 °C, and myosin molecules begin to form dimers and oligomers through head-to-head agglutination, mostly through disulfide bonds and hydrophobic contacts. When the temperature rises to around 40 °C, the myosin dimers and oligomers tend to assemble due to head-to-head contacts. The tail radiates into three-dimensional space, whereas the aggregation head is prone to aggregating into spherical masses. As the temperature continues to rise above 55 °C, the formed aggregates are crosslinked and aggregated by tail interactions to form particle units, mainly via hydrogen bonds, by forming a three-dimensional network structure [6,33,35].

The formation of the microstructure was mainly influenced by the relative rates of protein aggregation and unfolding, i.e., a compact and homogenous gel microstructure is usually formed while protein unfolding proceeds slower than its aggregation. Conversely, a coarse and heterogeneous gel microstructure is commonly obtained when the rate of unfolding exceeds that of aggregation [13,36]. Thus, it can be inferred that the freezing-induced denaturation of the myosin head was responsible for the reduced gelling properties of crayfish MPs.

### 3.5. UV Spectrum and FTIR

**Figure 5:**
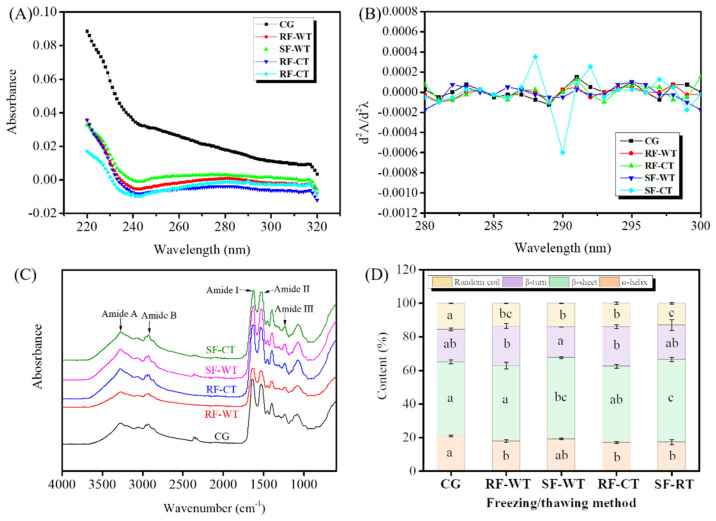
UV spectra (**A**), second-derivative UV spectra (**B**), FTIR spectrum (**C**), and protein secondary structures (**D**) of meat gels from crayfish subjected to different freeze-thawing treatments. CG, control group; RF, rapid freezing; SF, slow freezing; CT, cold thawing; WT, water immersing thawing. Different letters (a–c) in (**D**) for the content of protein secondary structures indicate significant differences (*p* < 0.05).

The UV spectra can be used to reflect the change of protein conformation by evaluating the microenvironment changes of aromatic amino acid side chains (such as tyrosine and tryptophan) [15]. As shown in Figure 5A, the maximum absorptions and positions of the peaks of UV spectra altered in the freeze-thawed groups, and it was suggested that the change of protein conformation in the meat gels of crayfish was responsible. Besides, the second derivative spectrum of crayfish meat gels was obviously altered in the SF-CT group (Figure 5B), revealing that the tertiary structure of gels obviously changed. The results of the UV spectrum further confirmed the alteration of protein conformation in gels induced by the freeze-thawing treatment, which was more pronounced by the SF-CT.

The FT-IR spectra for crayfish meat gels as influenced by freeze-thawing treatments are displayed in Figure 5C. The gels exhibited absorption bands at 3270 cm^−1^ (amide A, N–H or O–H stretching), 2920 cm^−1^ (amide B, C–H stretching), 1622 cm^−1^ (amide I, C=O and C=N stretching), 1521 cm^−1^ (amide II, C–N stretching and N–H bending), 1238 cm^−1^ (amide III, C–H bending), and 1071 cm^−1^ (C–O and C–C stretching) [14]. The migration of the amide A band could be used to estimate the interaction between protein and water molecules [37]. Commonly, the amide I band located at 1600–1700 cm^−1^ is often applied to estimate the information of protein secondary structures, including α-helix, β-turn, β-sheet, and random coil [14].

The secondary structures of the meat gels of crayfish were estimated, and the results are displayed in Figure 5D. The contents of α-helix of meat gels produced from the freeze-thawed crayfish decreased, while the contents of β-sheet increased, compared with the CG group. Additionally, it was also found that the SF led to a significant rise in the β-sheet content of crayfish gels (*p* < 0.05) at the expense of α-helix, presumably due to the disruption of hydrogen bonding in α-helix by large ice crystals during slow freezing. It has been reported that freeze-thaw treatments could promote the conversion of α-helix (ordered rigid structure) to a relatively loose β sheet (disordered flexible structure) in the MP of mirror carp (*Cyprinus carpio* L.), owing to the broken hydrogen bonds by physical effects as well as the protein oxidation accelerated by freeze-thawing [29]. Therefore, freeze-thawing not only affects the tertiary structure of crayfish meat gels, but also the secondary structure.

### 3.6. Digestibility, Antioxidant Activity, and SDS-PAGE

**Figure 6:**
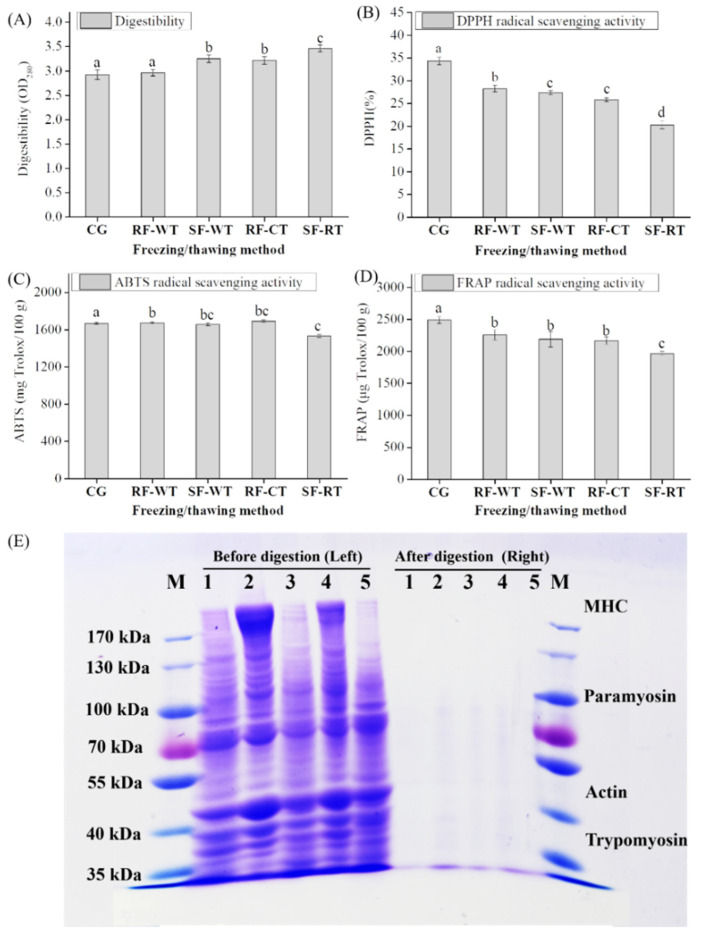
Digestibility (**A**), antioxidant activities (**B**–**D**), and proteins profiles (**E**) of meat gels from crayfish subjected to different freezing-thawing methods. Different letters (a–d) indicate significant differences (*p* < 0.05). Samples were prepared before (Left) and after (Right) digestion. M, marker; 1, CG; 2, RF-WT; 3, RF-CT; 4, SF-WT; 5, SF-CT. MHC, myosin heavy chain. CG, control group; RF, liquid nitrogen rapid freezing; SF, refrigerator slow freezing; CT, cold thawing; WT, water immersing thawing.

The effect of different freeze-thawing methods on the protein digestibility of crayfish meat gels by gastrointestinal environment simulation was shown in Figure 6A. SF before gelation of crayfish meat obviously enhanced the digestibility of the heat-induced gels (SF-WT vs RF-WT, SF-CT vs RF-CT) (*p* < 0.05). Meanwhile, the frozen crayfish thawed by CT showed a significant increase in protein digestibility of gels by comparison with WT (*p* < 0.05). The interference of SF and CT on digestion of crayfish meat gels could be mainly attributed to the denaturation or degradation of crayfish proteins, which altered the microstructure of the meat gels, increasing the accessibility of enzymes to hydrolytic sites [38].

For the antioxidant activity of the digest from crayfish meat gels, it was found that freeze-thawing treatments significantly decreased the DPPH-RSA, ABTA-RSA, and FRAP of the gels compared with the CG group (*p* <0.05), particularly for the SF-CT (Figure 6B–D). The lower antioxidant activity was probably related to the discrepant peptides derived from the crayfish meat gels [17]. Fang, et al. [39] reported that the proteolysis of surimi gels might be influenced by the structural properties under simulated gastrointestinal digestion, and they found more cross-linking in silver carp (*Hypophthalmichthys molitrix*) surimi gels induced by microbial transglutaminase (MTGase) retarded the pepsin digestion process at first, and the digestion sites and the quantities of peptides in the gels differed from the surimi gels without the addition of MTGase. However, other factors, such as molecular conformation, chemical bonds, etc., affecting the digestive characteristics (digestibility and produced peptides) of crayfish meat gels are still unclear.

The SDS-PAGE analysis was conducted to ascertain the protein patterns of crayfish meat gels before and after digestion. Before digestion, meat gel samples showed typical electrophoresis profiles, according to the report by Shao, et al. [40], the typical protein bands of gels of crayfish meat could be assigned to the myosin heavy chain (MHC, about 220 kDa), paramyosin (about 100 kDa), actin (about 44.3 kDa) and tropomyosin (about 36 kDa), respectively, and actin and MHC were the two main proteins. Notably, the MHC band intensity in the RF-WT and SF-WT groups (lanes 2 and 4 on the left) was lighter than that of the RF-CT and SF-CT (lanes 3 and 5 on the left), indicating that thawing by CT could lead to MHC degradation. However, after the digestion, these bands disappeared, and proteins were digested into peptides of less than 35 kDa. Therefore, further analytical methods, such as tricine-SDS-PAGE and mass spectrometry, need to be used to analyze the characteristics of these low molecular weight peptides.

### 3.7. Schematic Model

**Figure 7:**
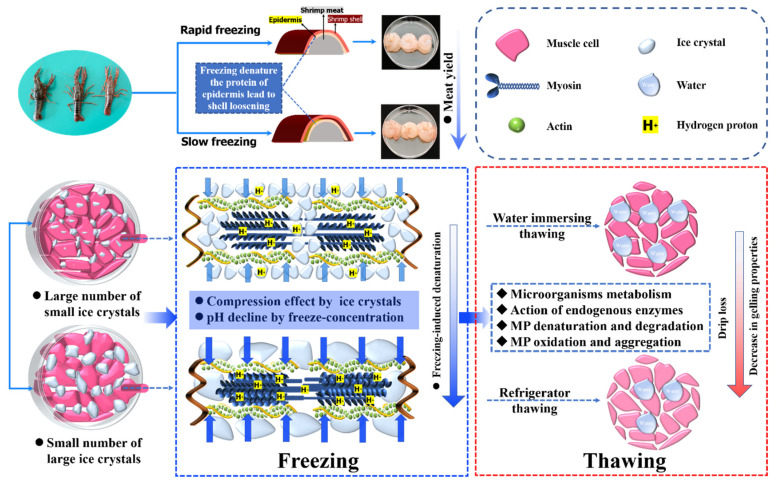
The schematic diagram of the effect of freeze-thawing on crayfish meat and its gelling properties.

Figure 7 shows the schematic model of the effect of freeze-thawing on crayfish meat and its gelling properties. Freezing denatures the protein of the epidermis, leading to shell loosening of crayfish, facilitating the subsequent peeling, and slow freezing causes greater protein denaturation and thus improves the meat yield [7]. Accordingly, freezing also disrupts muscle fibers and cells and induces the denaturation of MP [9,22]. Quick freezing produces a large number of small ice crystals distributed both intra- and extra-cellularly, while slow freezing generates a small number of large ice crystals that are extracellular [20]. Large ice crystals outside the cell can cause greater damage to myofibers and proteins. Freezing-induced denaturation of MP is mainly attributed to the compression effect of ice crystals and pH decline by freeze-concentration, which is more pronounced by using slow freezing [1,5]. The thawing process results in MP denaturation, oxidation, and aggregation, and even degradation, probably due to microorganism metabolism and the action of endogenous enzymes [13,30]. In the present study, CT was found to be disadvantaged in maintaining the gelling properties of MP for the frozen crayfish. We speculated that the denaturation of MP by freeze-thawing could change the relative rate of protein unfolding and aggregation during the subsequent heating.

## 4. Conclusions

RF greatly shortened the total freezing time compared with SF (490 min vs. 13 min), and WT obviously decreased the thawing time compared with CT. However, SF and CT increased the meat yield, probably due to the greater degree of protein denaturation. Different combinations of freezing-thawing resulted in the crayfish meat gels with various mechanical properties. A soft and flexible texture could be obtained by SF-CT, while a hard and flexible gel could be produced by RF-WT. Freeze-thawing not only affects the internal microstructure, but also the tertiary and secondary structures of protein in the crayfish meat gels. Moreover, those changes might increase protein digestibility but reduce the antioxidant activity of gels. These findings could provide a scientific basis for the manufacture of a novel gel-based meat product made from crayfish. The mechanism of freeze-thawing treatment on the gelling properties of MP remains to be further studied.

## Data Availability

Not applicable.
